# Characterisation of dust emissions from machined engineered stones to understand the hazard for accelerated silicosis

**DOI:** 10.1038/s41598-022-08378-8

**Published:** 2022-03-14

**Authors:** Chandnee Ramkissoon, Sharyn Gaskin, Leigh Thredgold, Tony Hall, Shelley Rowett, Richard Gun

**Affiliations:** 1 School of Public Health, Adelaide Exposure Science and Health, The University of Adelaide, Adelaide, SA Australia; 2 Mawson Analytical Spectrometry Services, School of Physical Sciences, The University of Adelaide, Adelaide, SA Australia; 3 Government of South Australia, SafeWork SA, 33 Richmond Road, Keswick, SA Australia

**Keywords:** Diseases, Respiratory tract diseases, Occupational health, Public health

## Abstract

Engineered stones are novel construction materials associated with a recent upsurge in silicosis cases among workers in the stonemason industry. In order to understand the hazard for the short latency of lung disease among stonemasons, we simulated *real-time* dust exposure scenario by dry-machining engineered stones in controlled conditions, capturing and analysing the *respirable* dust generated for physical and chemical characteristics. Natural granite and marble were included for comparison. Cutting engineered stones generated high concentrations of very fine particles (< 1 µm) with > 80% respirable crystalline silica content, in the form of quartz and cristobalite. Engineered stones also contained 8–20% resin and 1–8% by weight metal elements. In comparison, natural stones had far lower respirable crystalline silica (4- 30%) and much higher metal content, 29–37%. Natural stone dust emissions also had a smaller surface area than engineered stone, as well as lower surface charge. This study highlighted the physical and chemical variability within engineered stone types as well as between engineered and natural stones. This information will ultimately help understand the unique hazard posed by engineered stone fabrication work and help guide the development of specific engineering control measures targeting lower exposure to respirable crystalline silica.

## Introduction

Silicosis is an occupational lung disease commonly found in industries such as construction, metallurgy and coal and metal mining/quarrying. It is caused by the inhalation of respirable crystalline silica (RCS), in the form of quartz, tridymite or cristobalite^1^. Quartz, being the most abundant mineral in the Earth’s crust, is encountered more often than other polymorphs especially during occupational settings involving the mechanical processing of quartz-containing materials^2^. Occupational exposure to cristobalite can also occur in the ceramics industry as a result of quartz conversion in furnaces and in diatomaceous earth industries that process samples containing > 85% cristobalite^1,3^. Exposure to other polymoprhs of crystalline silica, such as coesite and stishovite, is rare^4^.

Engineered stones, also referred to as artificial stones, are novel construction materials commonly used for the fabrication of kitchen and bathroom countertops, floor and façade tiles. They owe their popularity to their durability, aesthetic appeal, variability and affordability. Their sales show no sign of slowing down; in fact, the U.S. market share are estimated to increase by 7.4% annually^5^. Unfortunately, the increased popularity of these new materials has been associated to the emergence of ‘accelerated silicosis’ among workers in the industry^6^. Tragically, the onset of silicosis has occurred following shorter exposure periods and shorter latency periods than traditionally seen^2^. A Spanish study reported increased cases of silicosis by 61% between 2007 and 2011^7^, which was a significant cluster within a small timeframe. The median age of workers diagnosed with silicosis was 33 years, following a median exposure to artificial stone dust of 11 years. Similar increases in silicosis incidence amongst workers have been reported in Israel, USA and Australia^5,6,8^.

The concern for engineered stone workers’ health stems from the fact that engineered stones typically contain > 90% quartz, joined together in a matrix with pigments and polymeric resins^9^. In comparison, natural stones contain much lower silica than artificial products. Marble and granite are two such natural stones containing 3% and 40% silica, respectively. Hence, fabrication processes such as cutting, drilling and polishing of engineered stones may result in high atmospheric concentrations of quartz-containing dust^10^. Interestingly, these mechanical processes are increasingly being carried out under wet conditions in industry, through the use of water-fed pneumatic grinders and polishers, to reduce dust exposure. Nevertheless, finishing tasks often end up being manual without water suppression, resulting in high potential for exposure to crystalline silica^11^.

The aetiology of accelerated silicosis in engineered stone workers is not well understood. Studies suggest that high exposure levels to crystalline silica during fabrication processes may be responsible for the rapid onset of silicosis^6^. The mechanism of silica toxicity is reliant on the small size of RCS, which can travel to the lower respiratory tract and gaseous exchange zones. The human body responds to the presence of these foreign particles by activating immune cells to engulf RCS particles through phagocytosis, inducing an inflammatory response; over time, sustained inflammation can lead to tissue damage and the development of fibrosis^12^. The engulfment of foreign particulate matter by macrophages also commonly leads to the generation of reactive oxygen species (ROS), which are important initiators of the fibrotic development^13^. It is important to note that these mechanisms explaining the association between silica inhalation and lung inflammation are consequences of long-term exposures to silica^12,13^. In the context of engineered stones, the onset of disease is relatively quick, making it unlikely that high exposures to silica alone could explain the extent of the damage; other chemical properties of the respirable dust should also be considered^14^. The chemical composition of resins and pigments, which would be an inherent part of the respirable emissions, could potentially also influence surface reactivity and pathogenesis^9,15^.

It is clear that the exposure science of engineered stones has knowledge gaps in terms of hazard characteristics of the freshly-generated respirable dust. In this view, we designed a study to explore the physical and chemical characteristics of respirable dust emissions generated by cutting twelve types of engineered stone. Three natural monolithic stones (white marble and white and black granites), subjected to similar processing methods, were included for comparison. This study provided a comprehensive assessment of the variability of the physicochemical properties of engineered stone emissions under realistic work exposure conditions.

## Results

The dust emitted by dry-cutting engineered stones in an enclosed environment was captured using respirable cyclones and subsequently subjected to various assays to determine their physical and chemical properties. Unless otherwise specified, all results pertain to the *respirable* fraction of machined engineered (and natural) stones. Respirable dust is defined as the fraction of inhalable dust that is able to penetrate the “unciliated airways” into the gas-exchange region of the lungs. They are typically dust of aerodynamic diameter < 4 µm^16,17^.

### Mineral composition

The engineered stone dust emissions were generally made of > 80% crystalline silica, often as a combination of quartz and cristobalite. Two engineered stones had only quartz in their composition (> 90%), while the majority of the other samples contained between 42 and 88% quartz. In engineered stone samples with relatively low (< 25%) quartz, such as ES6 and ES12, cristobalite accounted for the rest of the mineralogical composition (Table 1). Cristobalite was present in several other samples, albeit in lower concentrations than ES6 or ES12. It was present in moderate levels (36 ± 4.1%) in ES2, ES3 and ES11 and in low levels (< 5%) in ES1 and ES4 (Table 1). Compared to crystalline silica minerals, albite and rutile were less commonly found in respirable engineered stone dust. When present, they were observed in very low amounts, typically < 5% (Table 1). The only exception was ES4 which had a varied mineralogical composition, including 13% rutile (Table 1). No muscovite was observed in engineered stones.

**Table 1:** Physical and chemical properties of respirable dust emissions from machined engineered stones (ES1–12) and natural stones, namely black granite, white granite and white marble. The mineralogy of the stones was determined by X-Ray diffraction analysis, the resin content by thermogravimetric method and particle size and zeta potential by dynamic light scattering technique (water suspension, pH 7.4 at 25$^\circ$C). Results show average ± standard error (n = 3) and letters ^a-e^ indicate statistically significant differences using Duncan’s post hoc test (p < 0.05).

**Stone**	**Mineral content (%)**^**ϯ**^						**Resin content (%)**	**Particle size (nm)**	**Zeta potential (mV)**
	**α-Quartz**	**Cristobalite**	**Total RCS**^**†**^	**Albite**	**Rutile**	**Muscovite**			
ES1	86.7	4.20	90.9	0.52	–	–	8.62	630 ± 50 ^ab^	−26.1 ± 0.18 cd
ES2	42.4	44.0	86.4	1.18	0.43	–	12.0	533 ± 120 ^ab^	−32.9 ± 0.20 ^ab^
ES3	53.2	31.9	85.1	0.79	0.23	–	13.9	644 ± 69 ^ab^	−27.6 ± 4.60 ^ab^
ES4	67.8	2.62	70.4	1.38	12.6	–	15.6	500 ± 109 ^ab^	−28.2 ± 0.15 ^ab^
ES5	90.2	–	90.2	0.00	–	–	9.78	509 ± 30 ^ab^	−29.4 ± 0.82 ^abc^
ES6	20.0	65.5	85.5	1.02	–	–	13.5	417 ± 169 ^bc^	−25.7 ± 0.86 cd
ES7	86.7	–	86.7	0.00	–	–	13.3	416 ± 25 ^bc^	−30.0 ± 0.95 ^abc^
ES8	90.9	0.02	90.9	0.00	–	–	9.09	715 ± 91^a^	−30.0 ± 0.72 ^abc^
ES9	87.6	–	87.6	0.00	–	–	12.4	578 ± 44 ^ab^	−30. ± 0.62 ^abc^
ES10	87.6	–	87.6	0.28	–	–	12.1	218 ± 34 ^c^	−28.0 ± 0.70 ^bc^
ES11	46.4	31.4	77.8	5.85	–	–	16.4	576 ± 10 ^ab^	−33.8 ± 1.10 ^a^
ES12	25.4	54.7	80.1	0.00	–	–	20.0	455 ± 92 ^abc^	−26.6 ± 0.31 cd
Black granite	30.1	–	30.1	69.4	–	0.52		503 ± 11 ^ab^	−28.3 ± 0.55 ^bc^
White granite	3.5	–	3.50	–	–	–		634 ± 22 ^ab^	−15.2 ± 0.80 ^e^
White marble	11.0	–	11.0	–	–	–		575 ± 10 ^ab^	−22.9 ± 0.20 ^d^
Reference Quartz	96.7^§^	–	96.7	–	–	–		237 ± 32	−32.3 ± 2.80

^**ϯ**^The mineral composition was adjusted for the resin content of the stone.

^§^Certified quartz composition for the reference material was supplied by NIST.

^**†**^Calculated as the sum of α-quartz and cristobalite in the samples^25^.

The respirable dust emissions from natural stones had expectedly lower quartz content than engineered stones (Table 1). In decreasing order of quartz abundance were black granite (30%) > white marble (11%) > white granite (3.6%). The natural stones comprised several other minerals for example, albite, a feldspar mineral commonly found in igneous rocks such as black granite. White marble contained predominantly calcite (66%) and dolomite (22%) and white granite contained mostly dolomite (91%).

### Resin (organic content)

The resin content of the engineered stone dust emissions ranged from 8 to 20%. Three samples had low resin (< 10%), three other samples had between 10 and 15% resin, while fewer samples had high resin content (> 16%) (Table 1). Sample weight loss, as shown by a derivative thermogravimetric graph (DTG) (Supplementary Fig. S1), occurred in three stages: a small weight loss was observed while the sample was heated to up to ~ 300$^\circ$C, attributed to the desorption of water^9^; the second, and maximum, weight loss occurred at around 450$^\circ$C for all respirable engineered stone dust samples and was attributed to the loss of polymeric resin from the material. The third weight loss was observed at higher temperatures (~ 600$^\circ$C), but was considered minimal in comparison to the other two losses (Supplementary Fig. S1).

### Particle size distribution

In this study, the particle size of respirable dust generated by dry-cutting engineered and natural stones was analysed in a water suspension (pH 7.4 at 25$^\circ$C) using the dynamic light scattering (DLS) technique.

**Figure 1:**
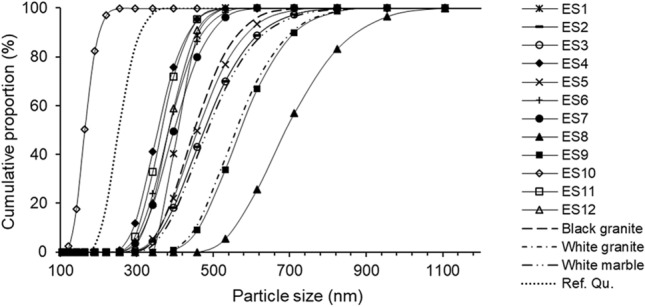
Representative cumulative plot for the particle size distribution of respirable dust generated from dry-cutting engineered (ES) (straight black lines) and natural stones, namely black granite, white granite and white marble (broken black lines). The reference quartz (Ref. Qu.; NIST 1878b) is illustrated as the black dotted line.

Dry-cutting engineered stones generated very fine particles, typically < 1 µm in size: > 90% of the dust particles had diameters in the size range of 190 nm to 825 nm (Fig. 1). The respirable dust emissions from cutting most engineered stones were similar in diameter, except for ES10 which had significantly finer dust, with particle diameter range of 142–295 nm (average 218 nm); in comparison, ES8 had the largest dust size with a particle diameter range of 459–1106 nm (average of 715 ± 91 nm) (Table 1, Fig. 1). Among all three natural stones, the black granite had a lower average particle size (503 nm) than the other two (534 and 675 nm respectively) (Table 1), but all three natural stones had particle size distributions comparable to those of engineered stones (Fig. 1).

The reference quartz was finer than all but one of the respirable engineered stone samples (Fig. 1). Similar results were observed by Pavan et al.^9^ , who reported artificial stone dust to be coarser and more heterogenous in size compared to a reference quartz sample.

### Zeta potential

The zeta potential of the stone emissions differed significantly within and between stone types (p < 0.01) (Table 1). The highest zeta potential was observed by ES11 (−33.8 ± 1.10 mV), while the majority averaged −29.9 ± 0.62 mV. Generally, engineered stone dust had significantly higher zeta potential than natural stone dust, with the exception of black granite (Table 1). With a zeta potential of −15.2 ± 0.80, the white granite emissions demonstrated the lowest charge among the stones studied. The reference quartz had a zeta potential of −32.3 ± 2.8 mV, which was comparable to engineered stones with high quartz (> 90%) content, such as ES8 (Table 1).

### Morphology

**Figure 2:**
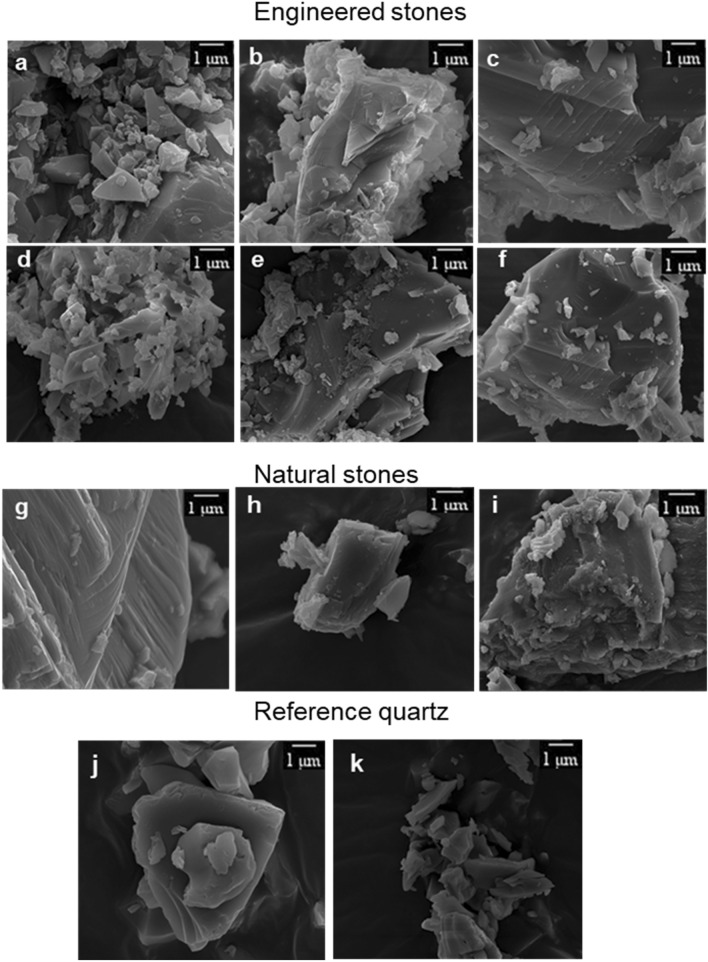
Representative SEM images of respirable dusts from machined engineered (**a**–**f**) and natural (**g**–**i**) stones, as well as reference quartz (**j**–**k**) under 40,000 and 20,000× magnification, which relate to 3 and 5 µm size fractions, respectively.

Engineered stone dust particles showed more irregular shapes with sharp edges and fractures along the surface (Fig. 2a–f), than natural stone dust particles, which exhibited natural layers with less conchoidal fractures on the surface (Fig. 2g–i). Compared to monolithics, engineered stone dust exhibited ‘charging’ and agglomeration of small particles, often attached to bigger particles, presumably by electrostatic forces^18^. The relative smaller size of the reference quartz was observed in the SEM images; the particles have smoother surfaces than stone that had undergone machining/dry-fracturing, particularly engineered stones (Fig. 2j–k).

### Specific surface area

Due to insufficient sample in the respirable fraction for the BET assay, specific surface area was assessed based on the *‘settled’* dust fraction generated by machining the stones. The specific surface area of the engineered stone dust was highly variable (range of 1.43 – 2.73 m^2^/g) and was higher than that of the natural stones (Supplementary Information Table S1). Five of the engineered stones had, on average, > 2.50 ± 0.13 m^2^/g surface area, while the rest averaged 1.72 ± 0.11 m^2^/g in surface area. In comparison, the specific surface area of the natural stones (range of 0.439 – 0.878 m^2^/g) was lower than the engineered stones (Supplementary Information Table S1).

### Elemental characterisation

**Figure 3:**
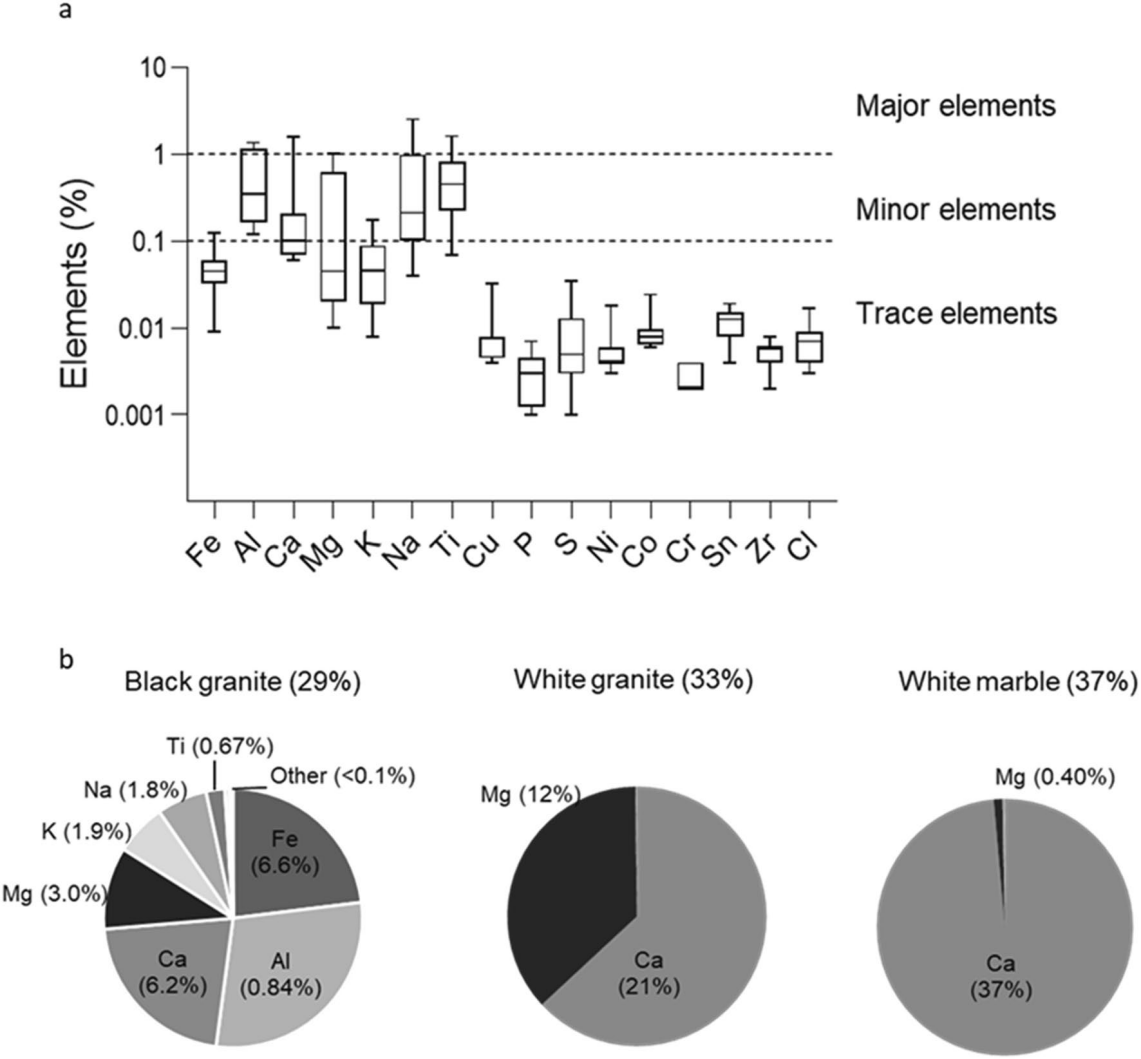
(**a**) Box-plot representation of the metal elemental composition (excluding Si) in twelve engineered stones. Centre line represents the median and whiskers represent the minimum and maximum values. (**b**) Variability of elements in the natural stones. The bracketed numbers in the pie charts show the abundance of elements as a percentage of the total elemental composition (reported in the chart titles). Elements not shown in the pie charts were below analytical detection limits.

The total elemental content (excluding Si) of the engineered stone dust samples varied from < 1% to 8% by weight. The majority of the samples had low elemental content (< 2%); two stone had 4%, while two had > 6% by weight elemental content (Fig. 3a).

Using the classification suggested by Di Benedetto et al.^15^ to identify trace (< 0.1% wt.), minor (< 1% wt.) and major (> 1% wt.) elements, it was observed that the following elements were in trace amounts in engineered stones: Cu, P, S, Ni, Co, Cr, Sn, Zr and Cl (Fig. 3a). Elements Fe, Ca, Mg, and K were predominantly in minor distributions. Certain elements such as Ca, Mg, Na and Ti had a range of concentrations from minor to major elemental fields.

Pearson’s correlation coefficients were used to assess whether the elements were correlated and regression analysis was used to determine whether the correlations were statistically significant. Elements such as Al, Ca and Mg were significantly correlated (r = 0.76; p < 0.01), suggesting a common origin for these metals in the engineered stone (Supplementary Information Table S2). The highest correlations were observed between Cu-Co (r = 0.96) and Ni-Co (r = 0.84), probably due to the association of Co and Cu on Ni laterite ores^19^.

The elemental content of natural stone emissions was much higher than that of the engineered stone emissions: 37% in white marble, 33% in white granite and 29% in black granite (Fig. 3b). The white marble and white granite contained predominantly alkaline metals as Ca and Mg, while the black granite had a more variable metal content, including transition metals such as Fe and Ti (Fig. 3b).

### Relationships between characteristics

To determine whether the parameters correlated to one another, multiple bivariate analyses were carried out on the following characteristics of the engineered stone: quartz, cristobalite, albite, rutile, specific surface area, particle size, zeta potential, metals content and total elemental content. No correlation among parameters was observed (r < 0.4; data not shown for brevity), suggesting that there was considerable variability in the characteristics of the emissions generated from dry-cutting engineered stones.

## Discussion

This study has focused on the characterisation of *respirable* dust following *real-time* generation from machined engineered stones, thus representing the most likely scenario facing workers in the engineered stone industry. The importance of analysing freshly-generated engineered respirable silica dust is apparent from the findings of Vallyathan et al.^20^, who demonstrated greater free radicals-induced oxidative damage potential from fresh milled quartz compared with aged quartz. To the best of our knowledge, Carrieri et al.^21^ reported the only other research for the characterisation of crystalline silica in the respirable fraction of dust generated by machining engineered stones (n = 3). We have, in this study, examined the characteristics of twelve engineered stones and three natural stones, with the aim of value adding to current literature about the properties of engineered stones which could shed light on their unique hazard potential.

In alignment with previous reports of the composition of respirable dust from machined artificial/engineered stones, the current engineered stone emissions consisted generally of > 80% by weight crystalline silica and 8–20% resin^21^. Further characterisation of the RCS was undertaken on the basis that the crystalline structure of the minerals may exert an influence on their toxicity^22^. In our study, 9 out of 12 engineered stone respirable dust samples had a combination of quartz and cristobalite structures, although quartz was still the dominant structure, forming > 55% of the total mineralogy. Cristobalite was the second most common mineral, while albite and rutile were detected in smaller amounts. Quartz and cristobalite differ from one another in their mineralogy, surface characteristics and natural association with other elements^23^. Early studies comparing the dose response of quartz and cristobalite on pulmonary function in rats showed that both structures were similarly detrimental to the lungs, although cristobalite elicited a slightly faster response than quartz^24^. However, subsequent animal experiments and epidemiological studies discounted these findings, by showing no evidence for differences in the inflammatory and fibrogenic potentials of quartz and cristobalite^23^. Horwell et al.^4^ even showed that cristobalite-rich volcanic ash was less toxic than expected and posed less of a respiratory health hazard than quartz. They attributed this finding to the relative open structure of cristobalite compared to quartz, which allows the substitution of cations such as aluminium (Al^3+^) and sodium (Na^+^) in the Si tetrahedral, hence affecting cristobalite toxicity^1,4^. Taken together, these studies show insufficient evidence that either mineral is more toxic than the other. Nonetheless, the high concentration of crystalline silica in the respirable dust from engineered stones may be cause for concern as quartz and cristobalite are the only crystalline silica minerals recognised as Group 1 carcinogens—“carcinogenic to humans”—by the International Agency for Research on Cancer^25^.

Apart from the mineral structure and content of engineered stone dust, the resin and metal/elemental composition were other properties that indicated their variability. The resin content of the respirable emissions for the engineered stones ranged from 8 to 20%, which was similar to the 5–15% polyester resin identified in artificial stone characterised by Carrieri et al.^21^. It has been suggested that resin can influence the reactive pathways of RCS, hence toxicity, in the lungs by acting as a ‘protective’ coating for the particles^9^. In their earlier studies, Pavan et al.^26^ subjected RCS particles to a thermal treatment to remove the polymeric resin and reported a significant increase in cytotoxicity, suggesting that resin partially covers the particle surface from interaction with cellular membranes.

There is a large body of research reporting short-term exposure to atmospheric particulate matter of < 2500 nm and associated adverse health effects especially on the respiratory system^27^. Dry-cutting engineered stones generated very fine particles of < 1200 nm, which have the capacity to penetrate deep into lung tissues^28^. The mean particle sizes of engineered stone samples, which ranged from 416 to 644 nm, were smaller than those recorded by Pavan et al.^9^, who measured mean diameters of dry-particles ranging from 1700 to 1900 nm, that is more than three times the diameter measured in our study. This disparity in particle size may be due to differences in the means of generation and collection of the dust: Pavan et al.^9^ collected deposited dusts in situ in engineered stone workspaces. In comparison, we actively collected the respirable fraction of dust generated from machined engineered stone. In another study by Carrieri et al.^21^ the respirable dust size analysis of an engineered stone sample (> 85% quartz) had a bimodal distribution, with one mode in the same range as in this study (~ 500 nm), whereas the other was in the ultrafine particle (UFP) range, commonly defined as particles < 100 nm^29^. Although visually observed, UFPs were not measured in the present study, likely due to the limitations of the air sampling or particle size analysis techniques. We are currently exploring some real-time measurement of UFP using direct reading instrumentation for more precise dust exposure assessment during engineered stone fabrication tasks.

It is important to hightlight the limitations in particle size analysis of the dust particles by the DLS technique. Whilst it is a widely accepted technique for determining particle size distribution, it may not be suitable for understanding composite samples generated during machining materials such as engineered stone. The technique relies on the assumption that all particles are homogenous and spherical in shape^30^. However, as evidenced by a high polydispersity index (> 0.7) and particle imaging by SEM, the dust particles in our study were, in fact, heterogenous in shape, size and structure. Apart from particle size and morphology, the surface properties of quartz have been reported to also play an important role in cytotoxicity, suggesting that the specific surface area of engineered stones may be a useful parameter for characterisation and differentiation between engineered and natural stones^26,31,32^.

Notwithstanding similar particle size distributions of the engineered and natural stone samples, we found a clear difference in specific surface area, which was greater in engineered stone. Microscopy imaging suggested that this difference was likely due to rough surfaces, cavities and conchoidal fractures at the surface of engineered stone particles; in comparison, natural stone dust particles exhibited smoother surfaces with clear natural layers. Within the engineered stone emissions, surface area varied considerably, from 1.43 to 2.72 m^2^/g (average 2.06 ± 0.13 m^2^/g), suggesting heterogeneity in the shape and size of the dust particles. The surface area of the engineered stone dust was generally higher than that reported Pavan et al.^31^ (~ 1.0 m^2^/g), which was expected as they also reported average greater sized dust particles in their study (~ 2000 nm). Increased surface area, associated with small particle size, for example UFPs, tend to be more toxic and cause more stress to alveolar macrophages than bigger particles^33^.

While high specific surface area of particles may itself confer toxic properties, it is possible that a high degree of specific reactivity of silica particles is also a contributing factor to its adverse health effects; in other words, small silica particles may have greater toxic effects than non-silica containing particles of comparable size. This is because quartz, particularly when fractured, has a very reactive surface as a result of the presence of chemically-active silanol groups (Si–OH)_2_)^34^. Early studies proposed that silanols may damage macrophages and induce inflammation but more recently, Pavan et al.^35^ identified a specific subfamily of silanols which are critical determinants of silica toxicity. They showed that the occurrence of specific patterns of silanols on the surface of quartz, the “nearly free silanols” (NFS), promote membranolysis and induce inflammation in rat lung cells. Their research challenged the original paradigm that crystallinity is key to silica toxicity^34,35^.

The surface reactivity of quartz, through its influence on surface charge, can be measured as the zeta potential in a specific medium and pH^22^. The zeta potential of the engineered stone dust emissions ranged from −25.7 ± 0.86 to −33.8 ± 1.10 mV and varied significantly among each other, highlighting the variability of fractured engineered stone surfaces. The zeta potential of two of the three natural stones was significantly lower than engineered stones. The exception was black granite, in which the zeta potential fell within the range of the engineered stone, albeit at the lower part of the range: this may be due to the greater silica content of the granite (30%) compared with the other natural stone samples (3.5% and 11%). An alternative explanation for the anomalous finding with black granite is its relatively high content of the metal elements Fe (6.6%) and Al (8.4%), compared with both the engineered stone and the other natural stone samples. Pavan et al.^22^ found that silica samples contaminated with metals reduced the level of negative zeta potential at certain pH levels; the authors stated that the zeta potential reflects the protonation state of the silanols on the surface of the silica crystals, which in turn determine the propensity to disrupt cell membranes. In this way zeta potential could therefore be used to predict the toxicity of a particulate^22,31^.

Cristobalite reportedly has a higher (more negative) zeta potential than quartz, especially at high pH, potentially due to its larger surface area from its tetragonal crystral structure, compared to quartz, which is trigonal^36^. In this set of samples, no correlation betweeen cristobalite content and zeta potential of engineered stone dust was observed (r < 0.5). Similarly, no correlations amongst any of the measured parameters were observed. It is likely that a bigger sample set of engineered stones, with more variable composition, is required to investigate the relationships among characteristics. Such information is essential when attempting to link engineered stone characteristics to pathogenesis.

Previous studies have reported engineered stone containing transition metals (e.g. Cu, Fe, Ti) as redox active species, as part of their pigments^9,15^. Our results build upon and are in good agreement with those reported by Pavan et al.^9^, whereby engineered stone mostly contained natural alkaline metals such as Ca, Mg, K and Al and elements such as Cu and Ni were in trace amounts. Titanium was the most variable transition metal element in engineered stone dust, ranging from trace (< 0.1%) to major (> 1%) quantities in the samples studied, possibly originating from the pigments and resins^37,38^. Although generally considered non-toxic, Ti (titanium dioxide, TiO_2_) has been shown to be an aetiological agent for lung inflammation, especially in the ultrafine fraction^39,40^. The possible role of metals in the toxicity of silica has been elicited before. For example, Clouter et al.^41^ (and references therein) suggested that the toxicity of quartz involves Fe. While the presence of Fe and Al has been considered for the potential reason for the differing zeta potentials of black granite and other natural stones, this could not explain the greater negative zeta potential of engineered stone compared to the black granite, since the concentration of Fe and Al is much lower in engineered stone. Several other elements not found in the natural stone samples were detected in the engineered stone ones, but only in trace quantities. Therefore, while we cannot exclude any role of metal ions in silica toxicity, it is unlikely that any such effect is mediated though the pathway linked with the generation of zeta potential.

This work has demonstrated that the dust emissions from machined engineered stones contain a high concentration of very fine particles that contain predominantly quartz and cristobalite, and therefore have the potential for a detrimental impact on respiratory health outcomes. This study showed the extent to which actively-generated *respirable* dust from engineered stones vary in their chemical properties, including crystalline silica, surface charge and resin content. In the present research, no correlation amongst the physicochemical properties of the engineered stone dust emissions was observed; further characterisation, in combination with clinical assays, could be useful to identify peculiar physical and chemical properties as biomarkers of pathogenesis. We also demonstrated that respirable dust emissions from natural stones were significantly different from engineered stone dust, with much lower silica content, smaller surface areas and generally lower surface charge. The results of this study shed light on the unique hazard posed by engineered stone fabrication work, and will help guide the development of specific engineering control measures targeting lower exposure to RCS.

## Materials and methods

### Samples

Twelve commercially-available engineered stones (ES1-ES12) were assessed in this study. These represented five brands/suppliers and were selected on the basis of their popularity amongst consumers (product sales volume nationally). Three natural stones, namely black granite, white granite and white marble, and a high purity reference quartz, SRM 1878b (National Institute of Standards and Technology, NIST, Maryland, US) were sourced for comparison.

### Sample generation and collection

The work practice of dry-cutting engineered and natural stones was simulated within a custom-made enclosed perspex cabinet (60 × 80 × 80 cm). The cabinet was fitted with two glove compartments and an air seal and the stones were held in place using clamps, in a similar way as described in Carrieri et al.^21^. An angle grinder (Metabo 720 W), fitted with a 105 mm diamond blade, was manually operated at ~ 10,000 rpm (Digital Tachometer QM1448, New England Instrument Co., NEIC) to make 3 mm wide zip cuts through the stone. A high-efficiency industrial vacuum (Metabo ASR 35 L ACP 1400 W) with flow rate 3660 l/min was connected to the cabinet to exhaust dust emissions.

Respirable dust was collected using either a Higgins-Dewell respirable cyclone (Casella Solutions, Maryland, USA) with 2.2 L/min flow rate or a Parallel Particle Impactors (PPI) respirable sampler (No.225–383, SKC Inc., Eighty Four, PA, USA) with flow rate 8.0 L/min. The respirable dust was collected on pre-weighed 25 mm and 37 mm PVC membrane filters (GLA-5000, SKC Inc., USA). Particle size and zeta potential analyses were carried out on freshly-generated dust (within 2 h of dust generation) to minimise risks of sample aggregation with time. For remaining characterisation assays all dust samples were stored in cool, dry conditions pending further assays.

### Sample characterisation

#### Mineral composition

The forms of crystalline silica in the engineered and the natural stones were determined by an X-ray Diffraction (XRD) technique conducted using a Bruker D8 Advance Powder X-ray Diffractometer (Bruker AXS Inc., Madison, Wisconsin, USA) with a Cu-radiation source operating at 40 kV and 40A, scanning 2 theta from 278 to 338 K with sample rotation of 30 rotations/min.

The respirable dust sample was transferred from the filter onto silicon wafers in a fine dusting over the centre of the wafer. Data was processed using Bruker DIFFRAC.EVA software and Crystallography Open Database reference patterns for identifying mineral phases. Quantification was calculated using TOPAS 4.2 software (Bruker AXS Inc., Madison, Wisconsin, USA).

#### Resin content

Proximate analysis of each stone was performed by thermogravimetric analysis (TGA), (Mettler-Toledo, Inc., TGA/DSC 2 STARe System, Columbus, Ohio, USA), using an ultra-microbalance to assess weight change upon pyrolysis. Samples (circa 5 mg) were heated from 30$^\circ$C to 1000$^\circ$C at a rate of 10$^\circ$C/minute under nitrogen atmosphere (flow rate 50 mL/minute).

#### Particle size and zeta potential

Stone dust samples were analysed for particle size and zeta potential on a Zetasizer Nano-ZS (Malvern Instruments Ltd., Worcestershire, UK) by light scattering techniques commonly used for nanoparticle suspensions characterisation^42^. The samples were suspended in MilliQ water (5 mg/10 mL), sonicated for 10 min at 50 Hz (Ultrasonic Cleaner FX8, Unisonics Pty. Ltd., Sydney, Australia) and diluted two-fold prior to size analysis. The pH of the solutions was measured to be 7.4 on a pH meter (Starter 300, OHAUS, New Jersey, USA). The zeta potential of the same suspensions (5 mg/10 ml) was assessed by the electrophoretic light scattering technique on the Zetasizer at 25$^\circ$C. The polydispersity index (PDI) of the dust suspensions was recorded.

The performance of the equipment for particle size and zeta potential analyses was assessed through the standard reference material of colloidal silica (ERM – FD 100, Geel, Belgium). Good agreement (within 10% variance) was observed between the reference and recorded values for colloidal silica. The measurements for particle size and zeta potential of the samples were done in triplicates.

#### Morphology

The morphology of generated dust particles was determined by Scanning Electron Microscopy (SEM) (FEI Helios Nanolab 600, USA), following loading of the sample (circa 1–2 mg) on double-sided adhesive tape and coating with platinum. The SEM images displayed were scaled using the ImageJ software (National Institutes of Health, Maryland, USA).

#### Specific surface area

Due to sampling limitations for respirable dust, specific surface area was determined on the total “settled” dust fraction (which included respirable), that was deposited in the chamber as the stones were machined. The samples were pre-weighed and degassed overnight at room temperature prior to analysis by the Brunauer–Emmett–Teller (BET) method on a surface area and porosity analyser (Micrometrics Tristar II 3020, Norcross, GA, United States), applying nitrogen as the adsorbate gas at −196$^\circ$C.

#### Elemental composition

Similar to the surface area assay, the determination of the metal elemental composition of the samples was carried out on the total ‘settled’ dust fraction by X-ray Fluorescence (XRF) at a commercial analytical laboratory, Bureau Veritas. No sample preparation was required; the stone dust samples were cast using a 12:22 lithium borate flux to form a glass bead. The content of Fe, Al_2_O_3_, MnO, TiO_2_, CaO, MgO, K_2_O, P, S, Na_2_O, Cu, Ni, Co, Cr, Pb, Zn, As, Sn, Sr, Zr, Ba, V, Cl was determined by XRF Spectrometry. The Si content of the dust samples was determined but not reported as part of the elemental composition.

### Statistical analysis

Any differences in the characteristics within stone samples were assessed for statistical significance at a 95% confidence level (p < 0.05) using Analysis of Variance (ANOVA). Duncan’s post hoc test identified statistical differences among samples. Bivariate correlation analyses measured the degree and strength of correlation, if any, between parameters.

## Supplementary Information

Supplementary Information.

## Data Availability

All data generated or analysed during this study are included in this published article (and its Supplementary Information files).
