# Supplementary material for: Characterisation of dust emissions from machined engineered stones to understand the hazard for accelerated silicosis
Source: Sci Rep. 2022 Mar 14;12:4351. doi: 10.1038/s41598-022-08378-8 (PMC8921240; doi:10.1038/s41598-022-08378-8)
Supplement: Supplementary file 1 — Supplementary Information. [file 41598_2022_8378_MOESM1_ESM.docx]

Characterisation of dust emissions from machined engineered stones to understand the hazard for accelerated silicosis

Chandnee Ramkissoon^1^, Sharyn Gaskin^1^*, Leigh Thredgold^1^, Tony Hall^2^, Shelley Rowett^3^ and Richard Gun^1^

^1^Adelaide Exposure Science and Health, School of Public Health, The University of Adelaide, South Australia

^2^School of Physical Sciences, The University of Adelaide, South Australia

^3^SafeWork SA, Government of South Australia, 33 Richmond Road, Keswick, South Australia

Corresponding author: [sharyn.gaskin@adelaide.edu.au](mailto:sharyn.gaskin@adelaide.edu.au) ORCID ID 0000-0002-1507-6425

Fig. S1: Thermogravimetric (TGA) (solid line) and derivative TGA (DTG) curves of an engineered stone sample (heating rate, 10⁰C min^−1^ to 1000⁰C under N_2_ atmosphere).

Table S1: Specific surface area of the ‘settled’ dust fraction (including respirable fraction) generated from machining engineered (ES1-ES12) and natural stone.

| Stone | Specific surface area |
| --- | --- |
|  | m^2^/g |
| ES1 | 2.72 |
| ES2 | 2.43 |
| ES3 | 2.73 |
| ES4 | 1.93 |
| ES5 | 1.62 |
| ES6 | 1.43 |
| ES7 | 1.78 |
| ES8 | 1.54 |
| ES9 | 1.87 |
| ES10 | 1.89 |
| ES11 | 2.34 |
| ES12 | 2.38 |
| Black granite | 0.768 |
| White granite | 0.878 |
| White marble | 0.439 |

Table S2: Pearson’s coefficients for the correlations between elements in engineered stone (n=12).

|  | Fe | Al | Ca | Mg | K | Na | Ti | Cu | P | S | Ni | Co | Cr | Sn | Zr |
| --- | --- | --- | --- | --- | --- | --- | --- | --- | --- | --- | --- | --- | --- | --- | --- |
| Al | 0.40 |  |  |  |  |  |  |  |  |  |  |  |  |  |  |
| Ca | 0.01 | 0.25 |  |  |  |  |  |  |  |  |  |  |  |  |  |
| Mg | 0.38 | 0.88** | 0.64** |  |  |  |  |  |  |  |  |  |  |  |  |
| K | 0.41 | 0.09 | 0.58 | 0.28 |  |  |  |  |  |  |  |  |  |  |  |
| Na | 0.16 | 0.62** | 0.91** | 0.90** | 0.46 |  |  |  |  |  |  |  |  |  |  |
| Ti | 0.23 | 0.64** | 0.17 | 0.64** | 0.01 | 0.61* |  |  |  |  |  |  |  |  |  |
| Cu | 0.02 | 0.30 | 0.26 | 0.26 | 0.07 | 0.30 | 0.18 |  |  |  |  |  |  |  |  |
| P | 0.22 | 0.43 | 0.13 | 0.10 | 0.21 | 0.10 | 0.07 | 0.29 |  |  |  |  |  |  |  |
| S | 0.12 | 0.74** | 0.13 | 0.61* | 0.22 | 0.42 | 0.72** | 0.00 | 0.17 |  |  |  |  |  |  |
| Ni | 0.12 | 0.74** | 0.13 | 0.61* | 0.22 | 0.42 | 0.72** | 0.00 | 0.17 | 0.08 |  |  |  |  |  |
| Co | 0.11 | 0.27 | 0.19 | 0.24 | 0.22 | 0.26 | 0.13 | 0.96** | 0.31 | 0.17 | 0.84** |  |  |  |  |
| Cr | 0.12 | 0.74** | 0.13 | 0.61* | 0.22 | 0.42 | 0.72** | 0.00 | 0.17 | 0.08 | 0.21 | 0.01 |  |  |  |
| Sn | 0.00 | 0.10 | 0.13 | 0.18 | 0.38 | 0.16 | 0.17 | 0.39 | 0.30 | 0.01 | 0.40 | 0.38 | 0.30 |  |  |
| Zr | 0.48 | 0.15 | 0.20 | 0.24 | 0.66** | 0.20 | 0.22 | 0.23 | 0.26 | 0.32 | 0.48 | 0.19 | 0.50 | 0.66** |  |
| Cl | 0.12 | 0.74** | 0.13 | 0.61* | 0.22 | 0.42 | 0.72** | 0.00 | 0.17 | 0.08 | 0.21 | 0.01 | 0.56 | 0.16 | 0.27 |

*: Statistical significance at 95% confidence level (p<0.05)

**: Statistical significance at 99% confidence level (p<0.01)
